# Functional Ultrasound Speckle Decorrelation‐Based Velocimetry of the Brain

**DOI:** 10.1002/advs.202001044

**Published:** 2020-07-26

**Authors:** Jianbo Tang, Dmitry D. Postnov, Kivilcim Kilic, Sefik Evren Erdener, Blaire Lee, John T. Giblin, Thomas L. Szabo, David A. Boas

**Affiliations:** ^1^ Neurophotonics Center Department of Biomedical Engineering Boston University Boston MA 02215 USA; ^2^ Biomedical Sciences Institute Copenhagen University Copenhagen 2200 Denmark

**Keywords:** brain imaging, cerebral blood flow velocity, field autocorrelation function, functional ultrasound velocimetry

## Abstract

A high‐speed, contrast‐free, quantitative ultrasound velocimetry (vUS) for blood flow velocity imaging throughout the rodent brain is developed based on the normalized first‐order temporal autocorrelation function of the ultrasound field signal. vUS is able to quantify blood flow velocity in both transverse and axial directions, and is validated with numerical simulation, phantom experiments, and in vivo measurements. The functional imaging ability of vUS is demonstrated by monitoring the blood flow velocity changes during whisker stimulation in awake mice. Compared to existing Power‐Doppler‐ and Color‐Doppler‐based functional ultrasound imaging techniques, vUS shows quantitative accuracy in estimating both axial and transverse flow speeds and resistance to acoustic attenuation and high‐frequency noise.

## Graphical abstract

A high‐speed, contrast‐free, quantitative ultrasound velocimetry (vUS) for blood flow velocity imaging throughout the rodent brain is developed based on the normalized first‐order ultrasound field signal temporal autocorrelation function. vUS is able to quantify blood flow velocity in both transverse and axial directions, and is capable of imaging blood flow velocity changes during whisker stimulation in awake mice.

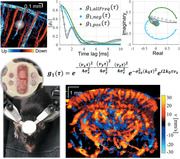

## 1 Introduction

Functional quantitative in vivo imaging of the entire brain with high spatial and temporal resolution remains an open quest in biomedical imaging. Current available methods are limited either by shallow penetration of optical microscopies that only allow imaging of superficial cortical layers, or by low spatiotemporal resolution such as functional magnetic resonance imaging (MRI) or positron emission tomography. Ultrasound‐based blood flow imaging techniques hold the promise to fulfill the unmet needs,^[^ ^1^, ^2^ ^]^ particularly with the emerging implementation of ultrafast ultrasound plane wave emission^[^ ^3^ ^]^ which paves the way for ultrasound to be applied for functional cerebral hemodynamic imaging of the entire rodent brain with 10–100 µm resolution.

Since the introduction of ultrafast plane wave emission‐based Power Doppler functional ultrasound imaging (PD‐fUS),^[^ ^4^ ^]^ an increasing number of studies are exploiting the capabilities of PD‐fUS for functional brain imaging studies.^[^ ^5^, ^6^, ^7^ ^]^ However, the exact relationship between the PD‐fUS signal and the underlying physiological parameters is quite complex as the PD‐fUS signal is also affected by the acoustic attenuation, beam pattern, clutter rejection and flow speed, in addition to the blood volume fraction and hematocrit.^[^ ^8^, ^9^ ^]^ On the other hand, ultrasound Color Doppler (CD‐fUS) is able to measure a specific physiological parameter of the axial blood flow velocity but suffers from unstable estimations of mean speed due to the presence of noise and from incorrect estimation if opposite flows exist within the measurement voxel.^[^ ^2^, ^4^, ^10^, ^11^, ^12^ ^]^ The microbubble tracking‐based ultrasound localization microscopy (ULM^[^ ^13^ ^]^) method is able to map the whole mouse brain vasculature (coronal plane) and quantify the in‐plane blood flow velocity (vULM^[^ ^13^, ^14^ ^]^) with ≈10 µm resolution. However, it suffers from a fundamental limitation of low temporal resolution as it requires extended data acquisition periods (≈150 s for 75 000 images^[^ ^13^ ^]^) to accumulate sufficient microbubble events to form a single vascular image and corresponding velocity map, limiting its potential for functional brain imaging studies.

Here, we report a novel ultrasound speckle decorrelation‐based velocimetry (vUS) method for blood flow velocity image of the rodent brain that overcomes the aforementioned limitations. We derived vUS theory which shows that the ultrasound field signal decorrelation in small vessels is not only determined by flow speed but also the axial velocity gradient and a phase term due to axial movement. We further developed a comprehensive experimental implementation and data processing methodology to apply vUS for blood flow velocity imaging of the rodent brain with high spatiotemporal resolution and without the need for exogenous contrast. We validated vUS with numerical simulations, phantom experiments, and in vivo measurements, and demonstrated the functional imaging ability of vUS by quantifying blood flow velocity changes during whisker stimulation in awake mice. We further show its advantage over PD‐fUS and CD‐fUS in terms of quantitative accuracy in estimating axial and transverse flow speeds and its resistance to acoustic attenuation and high frequency noise through phantom and in vivo measurements.

## 2 Results

### 2.1 vUS Theory

The time varying ultrasound signal detected from a measurement voxel at time *t* can be considered as the integration of all moving point scatters within the voxel, and the ultrasound pressure arising from a given voxel can thus be written as

(1) $$\begin{array}{c} sIQ\left(x_{0},y_{0},z_{0},t\right)=R\sum_{i_{s}}^{N_{s}}e^{-\frac{{\left(x_{i_{s}}\left(t\right)-x_{0}\right)}^{2}}{2\sigma_{x}^{2}}-\frac{{\left(y_{i_{s}}\left(t\right)-y_{0}\right)}^{2}}{2\sigma_{y}^{2}}-\frac{{\left(z_{i_{s}}\left(t\right)-z_{0}\right)}^{2}}{2\sigma_{z}^{2}}}e^{i2k_{0}\left(z_{i_{s}}\left(t\right)-z_{0}\right)} \end{array}$$

where, *sIQ* is the complex ultrasound quadrature signal of the moving particles of the voxel; *R* is the reflection factor; *i*_s_ is the index of the *i*th scatterer; *N*_s_ is the total number of scatterers within the voxel; ($x_{i_{s}}$, $y_{i_{s}},z_{i_{s}}$) is the position of the *i*_s_ scatter; (*x*_0_,*y*_0_,*z*_0_) is the central position of the measurement voxel; *σ*_*x*_, *σ*_*y*_, and *σ*_*z*_ are the Gaussian profile width at the 1/*e* value of the maximum intensity of the point spread function (PSF) in *x, y*, and *z* directions, respectively; and *k*_0_ is the wave number of the central frequency of the transducer. In Equation (1), we assumed that all scatter points have the same reflection factor.

**Figure 1:**
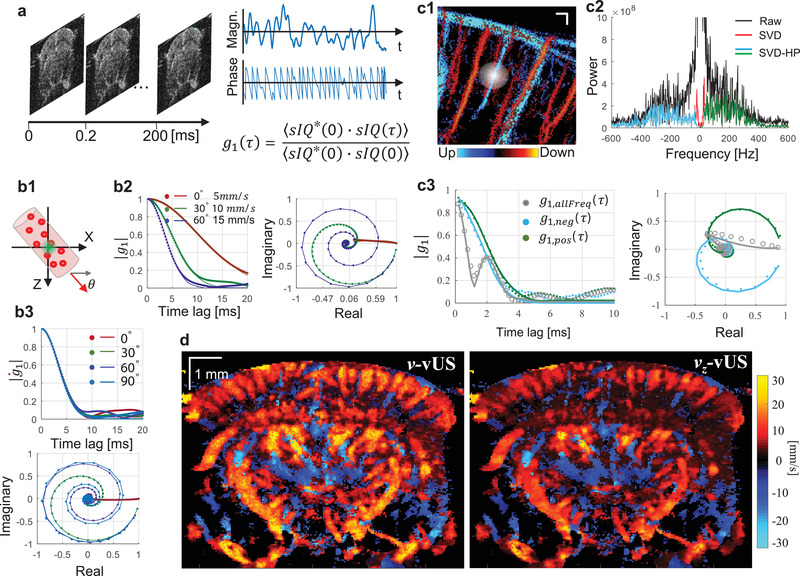
Principle of ultrasound field speckle decorrelation‐based velocimetry (vUS). a) A time series of a high frame rate complex ultrasound quadrature signal after bulk motion rejection (*sIQ*(*t*)) was used for *g*_1_(*τ*) calculation. b) Characteristics of *g*_1_(*τ*); b1) Scatterers flow through the measurement voxel at an angle *θ*; magnitude decorrelation of |*g*_1_(*τ*)| and field decorrelation of *g*_1_(*τ*) in the complex plane at b2) different angles with different speeds and b3) different angles with the same speed (*v*_0_ = s^−1^). c1) ULM measurement shows the microvasculature network in the brain; the white diffuse spot illustrates the ultrasound point spread function; c2) frequency power spectrum from in vivo data where descending and ascending vessels were observed in the same measurement voxel; c3) *g*_1_(*τ*) calculated using whole frequency signal (gray circles), negative frequency signal (cyan dots), and positive frequency signal (green dots), respectively. d) Representative total velocity map and axial velocity map reconstructed with vUS of a mouse brain; descending flow map is overlapped on the ascending flow map. The solid lines in (b) and (c) are the fitted *g*_1_(*τ*) using Equation (3).

As shown in **Figure** **1**a, the movement of particles will cause the detected ultrasound field signal to fluctuate in both magnitude and phase. This movement can be quantified based on the dynamic analysis theory of the normalized first‐order field temporal autocorrelation function (*g*_1_(*τ*)). *g*_1_(*τ*) of a time varying ultrasound signal for a measurement voxel is given by

(2) $$g_{1}\left(\tau \right)=E\left[\frac{{\left\langle sIQ^{*}\left(t\right)sIQ\left(t+\tau \right)\right\rangle }_{t}}{{\left\langle sIQ^{*}\left(t\right)sIQ\left(t\right)\right\rangle }_{t}}\right]$$

where *τ* is the time lag, *E*[…] indicates the average over random initial positions, 〈…〉_*t*_ represents an ensemble temporal average, *sIQ* is the clutter rejected ultrasound quadrature signal, and * is the complex conjugate. Figure 1b illustrates the major characteristics of *g*_1_(*τ*). Briefly, 1) *g*_1_(*τ*) decays faster for scattering particles flowing with higher speeds, 2) *g*_1_(*τ*) rotates and decays to (0, 0) in the complex plane, and 3) different flow angle has different decorrelation path in the complex plane, as shown in Figure 1b2. The rotating decorrelation in the complex plane is caused by the phase change due to axial movement. As shown in Figure 1b3, flows with the same total speed but in different angles have the same magnitude decorrelation (top panel) but different “rotation paths” in the complex plane (bottom panel). This feature gives *g*_1_(*τ*) analysis the ability to recover both axial velocity component and total flow speed.

When imaging the cerebral vasculature, the blood vessel diameter is usually less than the ultrasound system point spread function as indicated by Figure 1c1. In this case, the group velocity and velocity distribution must be taken into account as the relative movement of the scattering particles will result in additional decorrelation.^[^ ^15^ ^]^ To simplify the derivation, we used a Gaussian speed distribution where *v*_gp_ is the group velocity and *σ*_*v*_ describes the velocity distribution, and we finally arrive at

(3) $$g_{1}\left(\tau \right)=e^{-\frac{{\left(v_{x\mathrm{gp}}\tau \right)}^{2}}{4\sigma_{x}^{2}}-\frac{{\left(v_{y\mathrm{gp}}\tau \right)}^{2}}{4\sigma_{y}^{2}}-\frac{{\left(v_{z\mathrm{gp}}\tau \right)}^{2}}{4\sigma_{z}^{2}}}e^{-\sigma_{vz}^{2}{\left(k_{0}\tau \right)}^{2}}e^{i2k_{0}\tau v_{z\mathrm{gp}}}$$

From Equation (3), we note that in addition to flow speed, the axial velocity distribution *σ*_*vz*_ also contributes to the magnitude decorrelation, and the axial velocity component leads to a phase term in *g*_1_(*τ*) decorrelation. For details regarding the theoretical derivation, please refer to the Experimental Section‐vUS theory derivation.

In addition, we noticed from the in vivo data that it is common to have opposite flows present in the same measurement voxel when imaging the rodent brain, as shown in Figure 1c1. In this case, *g*_1_(*τ*) is a mix of dynamics of opposite flows and behaves very differently from that of the single direction flow as can be observed from Figs. 1b2 versus c3 (gray circles). In addition, we observed that the majority of the mouse cerebral blood vessels contain an axial velocity component to the flow. This axial flow component causes the frequency spectrum to shift to negative values if the flow is away from the transducer, and positive if the flow is toward the transducer. Thus, we used a directional filter (positive–negative frequency separation) method to obtain the positive frequency and negative frequency signals for the *g*_1_(*τ*) calculation, as shown in Figure 1c2.

To implement the vUS technology, we developed a comprehensive vUS data acquisition and processing method (Experimental Section—vUS Implementation, and Figure S1, Supporting Information). Figure 1d shows representative in‐plane total velocity and axial velocity maps of a mouse brain reconstructed by vUS. The descending flow velocity map which is reconstructed from the negative frequency component (*sIQ*_neg_) is overlapped on the ascending flow velocity map which is obtained from the positive frequency component (*sIQ*_pos_). Like the existing PD‐fUS and CD‐fUS techniques, vUS has an in‐plane spatial resolution of ≈100 µm which is determined by the ultrasound system acquisition parameters. Figure S2 (Supporting Information) shows more vUS results at different coronal planes.

### 2.2 Validation of vUS

**Figure 2:**
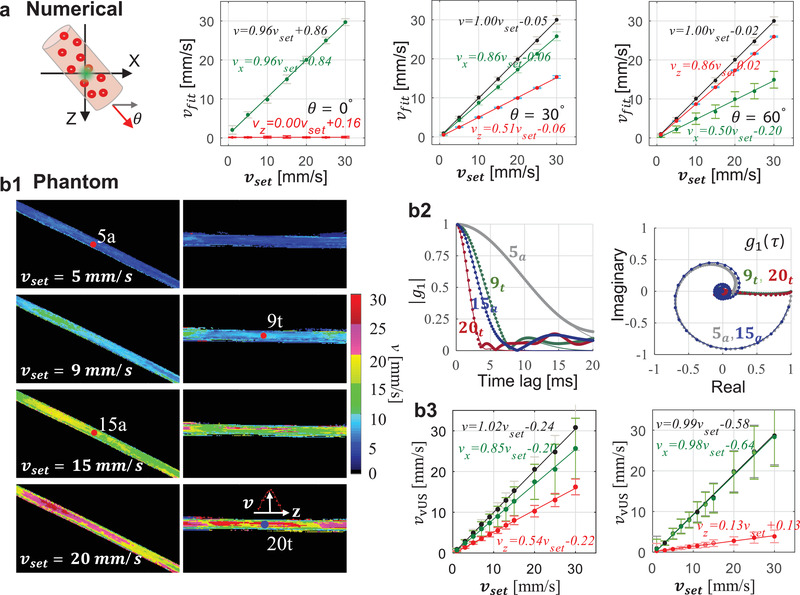
vUS numerical and phantom validation. a) Numerical simulation validation with different flowing angles and speeds. Error bars: standard deviation. b) Phantom validation of blood flowing through angled and transverse positioned microtubes (inner diameter 580 µm). b1) vUS reconstructed velocity maps of angled and transverse flows at different speeds. The inset in the right bottom panel shows the cross sectional laminar velocity profile of the transverse flow. b2) Experimental *g*_1_(*τ*) (dots) and corresponding vUS fit results (solid lines) for both angled and transverse flows at different speeds. b3) Results of vUS (*v*, *v_x_*, and *v_z_*) for transverse flow (*θ* ≈ 0°, left) and angled flow (*θ* ≈ 30°, right). Error bars: standard deviation.

The numerical simulation validation (details in the Experimental Section) results shown in **Figure** **2**a suggest that the vUS reconstructed total velocity (*v*), transverse velocity component (*v_x_*), and axial velocity component (*v_z_*) agree well with preset speeds and angles. It is worth noting that vUS is capable of measuring transverse flows (i.e., *θ*  =  0°) and differentiating the axial velocity component from the transverse velocity component for the angled flows, as shown by results from flow angle *θ*  =  30° and *θ*  =  60°. For all simulation results, the correlation coefficient between *v*_set_ and $v_{\mathrm{fit}\_\mathrm{mean}}$ were *r* > 0.99 with *p* < 0.001.

The phantom validation experiments (details in the Experimental Section) were performed with blood samples flowing through a microplastic tube buried within a static agarose phantom, as shown in Figure 2b. Figure 2b1 shows the velocity maps of both angled and transverse flows at preset speeds of 5, 9, 15, and 20 mm s^−1^. A laminar velocity profile was observed, particularly for higher flow speeds, as indicated in the inset of Figure 2b1. Figure 2b2 shows the experimental (dots) and vUS fitted *g*_1_(*τ*), from which we see that *g*_1_(*τ*) decays faster for higher speeds, and, as shown in the complex plane, *g*_1_(*τ*) rotates and decays to (0, 0) for angled flows (5_*a*_ and 15_*a*_) which is due to the axial velocity component inducing a phase shift as indicated in Equation (3). Different flow angles will have different “rotation paths” in the complex plane. Figure 2b3 shows the vUS reconstructed results compared to preset speeds, from which we note that the vUS measurements of total speed agree well with the preset speeds even for speeds as low as 1 mm s^−1^ for both transverse and angled flows. The correlation coefficient between *v*_set_ and $v_{\mathrm{fit}\_\mathrm{mean}}$ for transverse and angled flows were *r* > 0.99 with *p* < 0.001. Figure S3 (Supporting Information) presents all phantom experiment results obtained with the vUS, CD‐fUS, and PD‐fUS analysis methods.

**Figure 3:**
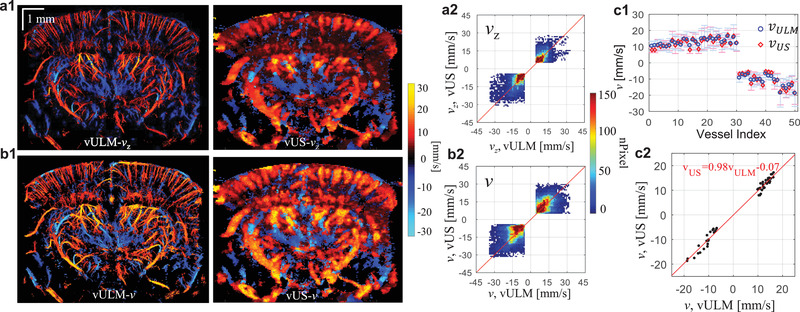
a,b) In vivo validation between vULM and vUS of axial velocity (a) and total velocity (b). (a2) and (b2) are pixel‐to‐pixel weighted scatter plot of common pixels of vULM and vUS with value |*v*| > 3 mm s^−1^. c1) Mean velocity and standard deviation measured with vULM (blue) and vUS (red) of 50 vessels marked in Figure S4a (Supporting Information). c2) Cross correlation of the mean total velocity of the 50 vessels between vULM and vUS (*r* = 0.984, *p* < 0.001).

We further performed in vivo validation by comparing the velocity measured with ultrasound localization microscopy velocimetry (vULM; see Experimental Section) against vUS, as shown in **Figure** **3**. We note that the measured axial velocity (Figure 3a1) and total velocity (Figure 3b1) agree well between vUS and vULM. The weighted scatter plots of all nonzero pixels between vUS and vULM in Figure 3a2,b2 indicate that the vUS measurement is highly correlated with the vULM measurement. We further compared the mean velocity of 50 vessels marked in Figure S4 (Supporting Information) between vULM and vUS. Figure 3c1 shows the mean velocity and standard deviation measured with vULM (blue) and vUS (red) of the 50 vessels. Figure 3c2 shows the scatter plot of the mean velocity of the 50 vessels measured with vULM and vUS. We note that the mean value of the 50 vessels agree well between vULM and vUS measurements with a linear relationship of $v_{z_{\mathrm{vUS}}}=0.98v_{z_{\mathrm{vULM}}}-0.07\;mm^{-1}$, indicating the accuracy of vUS for in vivo blood flow velocity imaging within the rodent brain.

### 2.3 vUS for Blood Flow Velocity Imaging in Response to Whisker Stimulation

**Figure 4:**
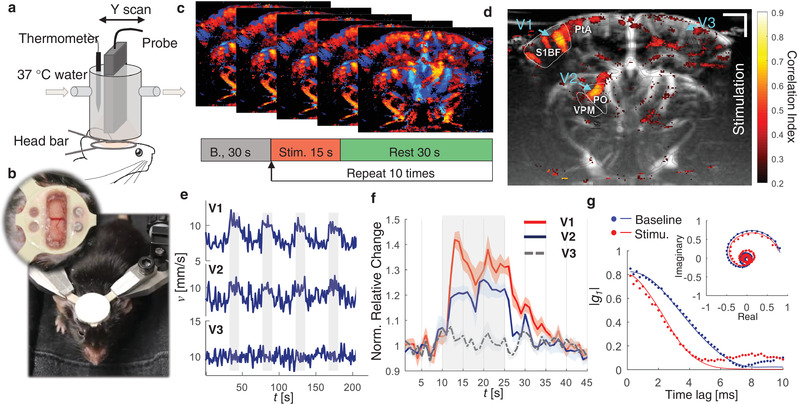
vUS of functional brain activation in awake mice. a) Experimental setup. b) Photos showing the trained mouse for awake‐head fixed ultrasound imaging; inset: a PMP film protected cranial window was prepared in the center of the head bar for ultrasound imaging. c) Whisker stimulation protocol and the vUS images were acquired at 1 frame s^−1^. d) Activation map in response to the mouse's left whisker stimulation. S1BF: primary somatosensory barrel field; PO: posterior complex of the thalamus; VPM: ventral posteromedial nucleus of the thalamus; PtA: posterior parietal association. The ROIs were identified according to Allen Mouse Brain Atlas(*16*). e) First four trials of blood flow velocity time course of vessels V1, V2, and V3 as marked in (d). The voxels of the three vessel ROIs were selected with absolute velocity value greater than 3 mm s^−1^. The gray shading indicates when stimulation was on. f) Average blood flow velocity relative change of the 10 trials for the three vessels. Error bar: standard error of the mean. g) Representative *g*_1_(*τ*) from baseline (blue) and under stimulation (red) for the same pixel within V1. Solid lines: vUS fitted *g*_1_(*τ*). Inset: *g*_1_(*τ*) in complex plane.

To demonstrate the functional imaging capability of vUS, we measured the blood flow velocity response to whisker stimulation. We developed an animal preparation protocol using a polymethylpentene (PMP) film^[^ ^6^ ^]^ with a custom designed headbar for chronic ultrasound imaging in awake mice (see Experimental Section), as shown in **Figure** **4**a,b. Following the published whisker stimulation protocol used in a previous PD‐fUS study,^[^ ^4^ ^]^ we used a stimulation pattern that consists of 30 s baseline followed by 10 trials of 15 s stimulation and with a 45 s interstimulus interval, as shown in Figure 4c. The vUS images were acquired at a rate of 1 frame s^−1^.

Figure 4d shows the correlation coefficient map between the blood flow velocity measured with vUS and the stimulation pattern. We note that in addition to the significant activation of vessels in the primary somatosensory barrel field (BF), the blood vessel flowing through the posterior complex (PO) and ventral posteromedial nucleus (VPM) of the thalamus also exhibited activation. Importantly, in addition to identifying significantly activated regions, vUS goes further and provides quantitative estimates of the evoked changes in the absolute flow velocity. The velocity time courses and velocity relative change averaged over the 10 trials of vessels V1 and V2 indicate robust blood flow velocity increases in response to the stimulation as shown in Figure 4e,f. The time course of vessel V3 on the ipsilateral cortex of the stimulation was plotted as a control region, which shows no correlation with the stimulation. The Video S1 (Supporting Information) shows the relative blood flow velocity changes of the whole recording. We further compared the *g*_1_(*τ*) for baseline and under stimulation of the same spatial pixel in V1, as shown in Figure 4g. It is evident that *g*_1_(*τ*) decays faster when under stimulation compared to that during the baseline, indicative of faster dynamics, i.e., elevated blood flow speed in response to whisker stimulation. The *g*_1_(*τ*) decorrelation function for the same measurement voxel in vessels of V1, V2, and V3 during baseline and whisker stimulation states are shown in Figure S5 (Supporting Information). The results suggest that *g*_1_(*τ*) decays faster during whisker stimulation in the responding vessels of V1 and V2, while in the control vessel of V3 the g1(*τ*) decorrelation rate does not change. Figure S6 (Supporting Information) shows more results of whisker stimulation experiments. Following the stimulation pattern commonly used in optical functional studies,^[^ ^16^ ^]^ we used vUS to detect the cerebral blood flow velocity change in response to a 5 s whisker stimulation with a 25 s interstimulus interval, as shown in Figure S6b (Supporting Information), and see that the measured blood flow velocity increases in response to the 5 s stimulation, indicating vUS is also sensitive to short duration stimulation evoked cerebral hemodynamic changes.

### 2.4 Comparison of vUS with PD‐fUS and CD‐fUS

**Figure 5:**
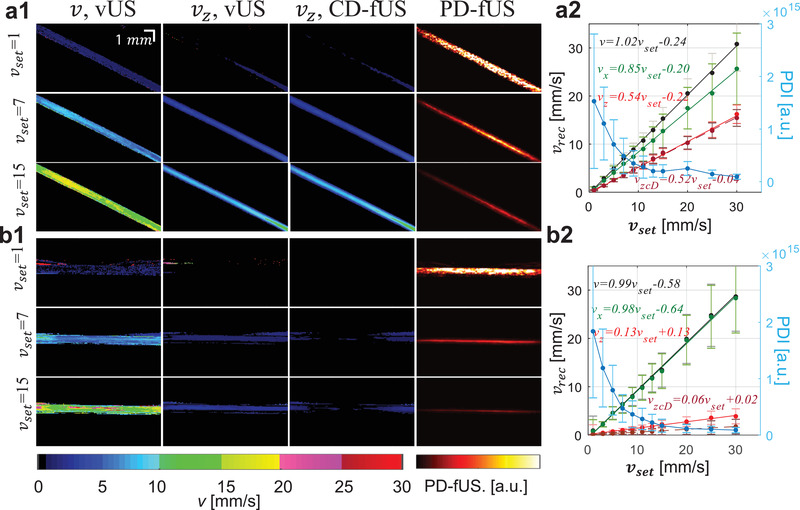
Phantom results comparison of vUS with Power‐Doppler‐based fUS (PD‐fUS) and Color‐Doppler‐based fUS (CD‐fUS). a,b) Angled (a) and transverse (b) flow phantom experiment results obtained with vUS (*v* and *v_z_*), CD‐fUS (*v_z_*), and PD‐fUS.

The data set acquired for the vUS calculation can also be used for PD‐fUS and CD‐fUS data processing, so there can be a direct comparison of the different approaches. The advantages of vUS processing are apparent as shown in **Figure** **5**. We see that: 1) CD‐fUS is only able to measure the axial velocity component (Figure 5a), 2) the signal intensity of PD‐fUS is not linearly related to total speed but nonlinearly decreases with increasing speed (Figure 5a2,b2), and 3) vUS is able to measure the blood flow velocity of both angled (Figure 5a) and transverse (Figure 5b) flows and differentiate the axial velocity component from the transverse velocity component (Figure 5a2), indicating the advantages of vUS in quantitatively imaging flow speeds in both axial and transverse directions.

**Figure 6:**
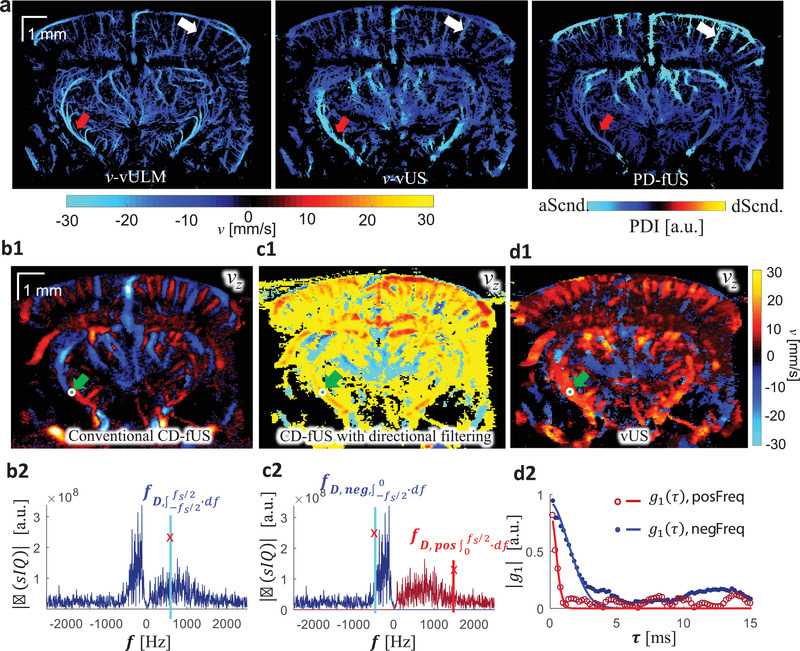
In vivo results comparison. a) In vivo ascending flow results obtained with vULM, vUS, and PD‐fUS, where vULM is used as the comparison standard and the ULM spatial mask was applied to both vUS and PD‐fUS. b1) Axial velocity (*v_z_*) map obtained with conventional CD‐fUS; b2) Doppler frequency (*f*_D_) is underestimated with conventional CD‐fUS. c1) Axial velocity map obtained with directional filtering‐based CD‐fUS; c2) Doppler frequencies (*f*_D, neg_ and *f*_D, pos_) are overestimated with the directional filtering‐based CD‐fUS. d1) Axial velocity map obtained with vUS; d2) *g*_1_(*τ*) calculated with positive frequency component and negative frequency component after directional filtering; dots: experimental data; solid line: theoretical fitting. Descending flow velocity maps were overlapped on ascending flow velocity maps in (c1) and (d1).

**Figure** **6**a compares the in vivo measurements of ascending flow (positive frequency component) obtained with vUS and PD‐fUS. Using the vULM measurement as the comparison standard of flow velocity, we note that vUS agrees well with vULM, while PD‐fUS has high signal intensity in superficial layers and low signal intensity in deep regions, as indicated by the white and red arrows, indicating the strong dependence of the PD‐fUS signal on acoustic attenuation. In contrast, vUS is not affected by acoustic attenuation as the normalization processing cancels the heterogeneous acoustic distribution. Figure 6b1 shows the axial velocity maps obtained with conventional CD‐fUS^[^ ^4^ ^]^ (Methods, Supporting Information). The conventional CD‐fUS suffers from underestimation of Doppler frequency (*f*_D_) due to mutual frequency cancellation when opposite flows exist within a measurement voxel, as illustrated in Figure 6b2. For a fair comparison between vUS and the Doppler methods, we applied CD‐fUS processing on the directional filtered data that we used for vUS processing. As shown in Figure 6c, we note that the blood flow speed is overestimated by the directional filtering‐based CD‐fUS. This overestimation happens because of high frequency noise causing overestimation of the Doppler frequency (*f*_D_) when a directional filter is applied and thus a higher speed bias, as shown in Figure 6c2. In comparison, vUS does not suffer from the high‐frequency noise as the high‐frequency noise is uncorrelated and only causes *g*_1_(*τ*) to drop to a lower value at the first time lag but it does not affect the decorrelation rate of *g*_1_(*τ*) at longer time lags, which is determined by the correlated motion of flowing red blood cells, as shown in the bottom panel of Figure 6d2. Thus, by fitting the decorrelation of *g*_1_(*τ*) the blood flow velocity can be accurately reconstructed by vUS, as shown in Figure 6d1.

## 3 Discussion

The development of robust blood flow velocity measurement technologies has been of great importance in neuroscience research as quantifying blood flow alterations enables the assessment of brain disease^[^ ^17^, ^18^, ^19^ ^]^ and interpretation of regional neural function according to neurovascular coupling.^[^ ^20^ ^]^ In this work, we introduced vUS based on the first‐order temporal field autocorrelation function analysis of the ultrasound speckle fluctuations to quantify cerebral blood flow velocity with a temporal resolution of 1 frame s^−1^ (up to 5 frames s^−1^ in theory), with a greater than 10 mm penetration depth, and ≈100 µm spatial resolution. vUS provides much deeper penetration compared to optical velocimetry methods which are usually restricted to superficial layers of less than 1 mm depth^[^ ^21^ ^]^ while maintaining high spatial and temporal resolution compared MRI‐based phase contrast velocity mapping.^[^ ^22^ ^]^

Using ultrasound signal decorrelation analysis to estimate flow speed dates back to the 1970s. Atkinson and Berry^[^ ^23^ ^]^ have shown that the motion of moving scatterers is encoded in the fluctuations of the ultrasound signal and Bamber et al.^[^ ^24^ ^]^ demonstrated that the ultrasound signal decorrelation could be used to image tissue motion and blood flow. Wear and Popp and others^[^ ^8^, ^9^, ^25^, ^26^, ^27^, ^28^ ^]^ showed that the decorrelation of ultrasound signal decays following a Gaussian form. In this paper, we showed that the ultrasound signal field decorrelation is governed by three terms, including the flow speed, the gradient of the axial velocity, and an axial‐velocity‐dependent phase term. This phase term gives vUS the ability to differentiate the axial velocity component from the transverse velocity component.

The high‐frame‐rate ultrafast ultrasound plane‐wave emission and acquisition paves the way for vUS implementation, which permits the speckle decorrelation caused by the moving scattering particles to be resolved with sufficiently high temporal resolution required to capture the speckle decorrelation within the small measurement voxels. The combination of spatiotemporal singular value decomposition and high pass filtering plays an important role in rejecting bulk motion which enables the decorrelation of *g*_1_(*τ*) to represent the dynamics of the motion of red blood cells and to not be confounded by bulk motion. For blood flow velocity imaging of the brain, vUS reconstructs both descending and ascending flow velocities from the negative frequency component and positive frequency component by applying directional filtering, respectively. We further developed a comprehensive fitting algorithm to reconstruct axial and transverse blood flow velocities. The proposed vUS technique was validated with numerical simulation, phantom experiments, and in vivo blood flow velocities obtained with vULM. The functional whisker stimulation experiment result agrees with previous rodent functional studies that mechanoreceptive whisker information reaches the barrel cortex via the thalamic VPM nuclei,^[^ ^29^ ^]^ and the PO is a paralemniscal pathway for whisker signal processing.^[^ ^30^ ^]^ This experiment demonstrates that vUS is sensitive to quantify the cerebral blood flow velocity change in response to functional stimulation and can be applied for brain imaging in awake mice.

Compared to PD‐fUS, vUS is a quantitative imaging modality for assessing blood flow velocity while the PD‐fUS signal decreased with increasing speed and is strongly affected by the acoustic attenuation. Compared to CD‐fUS, vUS is able to measure both axial and transverse flow velocities and is resistant to high frequency noise compared to the directional filtering‐based CD‐fUS which suffers from large or random values in regions with a low signal‐to‐noise ratio. Compared to vULM, vUS has lower spatial resolution but has much higher temporal resolution (up to 5 Hz of vUS compared to 2 min per frame of vULM) and is applicable for awake functional studies in rodents requiring high temporal resolution. In addition, it measures the flow velocity of the intrinsic contrast of red blood cells while vULM measures the speed of microbubbles. One important application that will be enabled by the absolute blood flow velocity measured with vUS is that the metabolic rate of oxygen can be quantitatively estimated if vUS measurements are combined with quantitative oxygenation measurements using multispectral photoacoustic tomography,^[^ ^31^, ^32^ ^]^ providing a new high resolution biomarker for neuroscience research.

A limitation is that vUS is not sensitive to measuring blood flow velocity in small vessels with low flow speeds due to the use of the spatiotemporal filter which rejects slow dynamics from the signal. Also, limited by the spatial resolution of the ultrasound system, the reconstructed blood flow velocity of a measurement voxel may represent integrated dynamics of multiple vessels that flow through the measurement voxel. For the results presented in this work, vUS was simplified to estimate in‐plane 2D velocities (i.e., *v_x_* and *v_z_*), ignoring decorrelation from flow in the y‐direction (see Experimental Section for justification). This simplification, however, results in a moderate overestimation of the transverse velocity (*v_x_*) as *v_x_* tends to compensate for the decorrelation caused by *v_y_*. Nevertheless, we note that the measured total velocity is very close to that obtained with vULM as shown in Figure 3. In the future, with the development of fast 3D ultrasound imaging technology using a 2D transducer matrix, vUS can be easily adopted for 3D velocimetry of the whole rodent brain.

## 4 Experimental Section

### 4.1 vUS Theory Derivation

The complex ultrasound quadrature signal of particles moving at the same speed in a measurement voxel can be written as

(4) $$\begin{array}{c} sIQ\left(x_{0},y_{0},z_{0},t\right)=R\sum_{i_{s}}^{N_{s}}e^{-\frac{{\left(x_{i_{s}}\left(t\right)-x_{0}\right)}^{2}}{2\sigma_{x}^{2}}-\frac{{\left(y_{i_{s}}\left(t\right)-y_{0}\right)}^{2}}{2\sigma_{y}^{2}}-\frac{{\left(z_{i_{s}}\left(t\right)-z_{0}\right)}^{2}}{2\sigma_{z}^{2}}}e^{i2k_{0}\left(z_{i_{s}}\left(t\right)-z_{0}\right)} \end{array}$$

Considering the basic scenario that all scatters have identical dynamics, i.e., the scatters are moving in the same direction with same speed, the ultrasound pressure of the resolution voxel at time lag *τ* can be written as

(5) $$\begin{array}{ccc} sIQ(x_{0},y_{0},z_{0},t+\tau ) & = & R\sum_{i_{s}}^{N_{s}}e^{-\frac{{\left(x_{i_{s}}\left(t\right)+v_{x}\tau -x_{0}\right)}^{2}}{2\sigma_{x}^{2}}-\frac{{\left(y_{i_{s}}\left(t\right)+v_{y}\tau -y_{0}\right)}^{2}}{2\sigma_{y}^{2}}-\frac{{\left(z_{i_{s}}\left(t\right)+v_{z}\tau -z_{0}\right)}^{2}}{2\sigma_{z}^{2}}}\\ & & e^{i2k_{0}\left(z_{i_{s}}\left(t\right)+v_{z}\tau -z_{0}\right)} \end{array}$$

According to Equation (2), *g*_1_(*τ*) for particles flowing identically within the ultrasound measurement voxel can be derived to be

(6) $$g_{1}\left(\tau \right)=e^{-\frac{v_{x}\tau^{2}}{4\sigma_{x}^{2}}-\frac{v_{y}\tau^{2}}{4\sigma_{y}^{2}}-\frac{v_{z}\tau^{2}}{4\sigma_{z}^{2}}}\;e^{i2k_{0}v_{z}\tau }$$

For microvasculature imaging of the rodent brain, the group velocity and velocity distribution must be taken into account as the relative movement of scatters will result in additional decorrelation. To simplify the derivation, a Gaussian distributed velocity model was used to describe the velocity distributed flow

(7) $$P\left(v_{x},v_{y},v_{z}\right)=\frac{1}{\pi \sqrt{\pi }\sigma_{vx}\sigma_{vy}\sigma_{vz}}e^{-\frac{{\left(v_{x}-v_{x\mathrm{gp}}\right)}^{2}}{\sigma_{vx}^{2}}-\frac{{\left(v_{y}-v_{y\mathrm{gp}}\right)}^{2}}{\sigma_{vy}^{2}}-\frac{{\left(v_{z}-v_{z\mathrm{gp}}\right)}^{2}}{\sigma_{vz}^{2}}}$$

where *P*(*v_x_*, *v_y_*, *v_z_*) is the velocity distribution probability; *v*_gp_ is the group velocity; and *σ*_*v*_ describes the velocity distribution.

*g*_1_(*τ*) for the Gaussian speed distribution flow is derived to be

(8) $$\begin{array}{ccc} g_{1}\left(\tau \right) & = & \sqrt{\frac{64\sigma_{x}^{2}\sigma_{y}^{2}\sigma_{z}^{2}}{\left(4\sigma_{x}^{2}+\sigma_{vx}^{2}\tau^{2}\right)(4\sigma_{y}^{2}+\sigma_{vy}^{2}\tau^{2})\left(4\sigma_{z}^{2}+\sigma_{vz}^{2}\tau^{2}\right)}}\\ & & e^{-\frac{{\left(v_{xgp}\tau \right)}^{2}}{4\sigma_{x}^{2}+\sigma_{vx}^{2}\tau^{2}}-\frac{{\left(v_{ygp}\tau \right)}^{2}}{4\sigma_{y}^{2}+\sigma_{vy}^{2}\tau^{2}}-\frac{{\left(v_{zgp}\tau \right)}^{2}+4\sigma_{z}^{2}\sigma_{vz}^{2}{\left(k_{0}\tau \right)}^{2}}{4\sigma_{z}^{2}+\sigma_{vz}^{2}\tau^{2}}}e^{i2k_{0}\tau \frac{4\sigma_{z}^{2}v_{zgp}}{4\sigma_{z}^{2}+\sigma_{vz}^{2}\tau^{2}}} \end{array}$$

From the observations, the typical decorrelation time (*τ*_c_) for blood flow with a speed around 10 mm s^−1^ is ≈5 ms. Therefore, $\sigma_{v↔}^{2}\tau^{2}<6.25\times 10^{-4}\;mm^{2}$ which is more than eight times smaller than $4\sigma_{↔}^{2}\geq 50\times 10^{-4}\;mm^{2}$, where “↔” represents the coordinate direction (i.e., *x, y*, or *z*). Thus, the theoretical equation of *g*_1_(*τ*) can be further simplified to be

(9) $$g_{1}\left(\tau \right)=e^{-\frac{{\left(v_{x\mathrm{gp}}\tau \right)}^{2}}{4\sigma_{x}^{2}}-\frac{{\left(v_{y\mathrm{gp}}\tau \right)}^{2}}{4\sigma_{y}^{2}}-\frac{{\left(v_{z\mathrm{gp}}\tau \right)}^{2}}{4\sigma_{z}^{2}}}\;e^{-\sigma_{vz}^{2}{\left(k_{0}\tau \right)}^{2}}e^{i2k_{0}\tau v_{z\mathrm{gp}}}$$

where, *σ*_*x*_, *σ*_*y*_, and *σ*_*z*_ are the Gaussian profile width at the 1/*e* value of the maximum intensity of the point spread function (PSF) in *x, y*, and *z* directions, respectively; *v*_gp_ is the group velocity; *σ*_*vz*_ describes the axial velocity distribution; and *k*_0_ is the wave number of the central frequency of the transducer.

### 4.2 vUS Implementation

#### 4.2.1 Coherent Plane Wave Compounding‐Based Data Acquisition

The ultrasound signal was acquired with a commercial ultrafast ultrasound imaging system (Vantage 256, Verasonics Inc. Kirkland, WA, USA) and a linear ultrasonic probe (L22‐14v, Verasonics Inc. Kirkland, WA, USA). The Vantage 256 system had 256 parallelized emission and receiving channels, and could acquire planar images at a frame rate up to 30 kHz when the imaging depth was ≈15 mm. The L22‐14v ultrasonic probe had 128 transducer elements with a pitch of 0.1 mm and a center frequency of 18.5 MHz with a bandwidth of 12.4 MHz (67%, −6 dB). It had an elevation focus at *z* = 6 mm.

To ensure sufficient temporal resolution, the ultrasound plane wave frame rate was set to 30 kHz which was mainly limited by the transmit time of the ultrasound signal in the sample through the intended imaging depth, as shown in Figure S1a (Supporting Information). To enhance the signal‐to‐noise ratio while preserving sufficient temporal resolution, coherence plane wave was further employed compounding^[^ ^33^ ^]^ at five emitting angles (−6°, −3°, 0°, 3°, 6°) to form a compounded image whose frame rate was 5 kHz, as shown in Figure S1b (Supporting Information).

In addition, to acquire sufficient ensemble averaging of the US speckle fluctuations for the vUS analysis, 200 ms of data were acquired, i.e., 1000 compounded images, to calculate *g*_1_(*τ*) over a range of 0 ms < *τ* <20 ms. Therefore, the maximum vUS frame rate was 5 frames s^−1^. However, for extended data acquisition (i.e., >1 min), the maximum vUS frame rate was reduced to 1 frame s^−1^ due to limited data transfer and saving requirements.

#### 4.2.2 Clutter Rejection

For the phantom data processing, a spatiotemporal filtering method (singular value decomposition, SVD, Equation 10 ^[^ ^34^ ^]^) was used to remove the first two (*Nc* = 3) highest singular value signal components. To reject the bulk motion signal from the in vivo data, a combination of SVD and high pass filtering was used. The first 20 highest singular value signal components were removed (*Nc* = 21), followed by a fourth‐order Butterworth high pass filtering with a cutoff frequency of 25 Hz corresponding with a 1 mm s^−1^ speed cutoff.

(10) $$sIQ=\sum_{i=Nc}^{N}S\left(z,x\right)\lambda_{i}V\left(t\right)$$

where *sIQ* is the dynamic signal, *Nc* is the cutoff rank for SVD processing, *S*(*z*, *x*) is the spatial singular matrix, *λ*_*i*_ is the singular value corresponding with the *i*th rank, and *V*(*t*) is the temporal singular vector.

#### 4.2.3 vUS Fitting Algorithm

Figure S1d (Supporting Information) summarizes the vUS data processing algorithm. Based on the developed vUS theory for in vivo brain imaging, the clutter rejected *sIQ* data of a measurement voxel, *sIQ*(*z*, *x*), were first directionally filtered to obtain the negative frequency signal component (descending flow) and the positive frequency signal component (ascending flow) using the directional filtering processing (Equations 11 and 12)

(11) $$F\left(sIQ\right)=F_{\mathrm{neg}}\left(sIQ\right)+F_{\mathrm{pos}}\left(sIQ\right)$$

(12) $$sIQ_{\mathrm{neg}}=F^{-1}\left[F_{\mathrm{neg}}\left(sIQ\right)\right],\;sIQ_{\mathrm{pos}}=F^{-1}\left[F_{\mathrm{pos}}\left(sIQ\right)\right]$$

where *sIQ*_neg_ and *sIQ*_pos_ are the complex ultrasound quadrature signal of the negative frequency and positive frequency, respectively; F denotes the Fourier transform; and $F^{-1}$ denotes the inverse Fourier transform. $g_{1_{\mathrm{neg}}}\left(\tau \right)$ and $g_{1_{\mathrm{pos}}}\left(\tau \right)$ for *sIQ*_neg_ and *sIQ*_pos_ are obtained using Equation (2), respectively.

Criteria including the ratio of positive/negative frequency power to whole frequency power (Equation 13) and the absolute value of *g*_1_(*τ*) at the first time lag (Equation 14) were used to control signal quality for data processing

(13) $$\begin{array}{ccc} R_{\mathrm{pos}} & = & \frac{\sum F{\left(sIQ\right)}_{f>0}}{\sum F{\left(sIQ\right)}_{\mathrm{all}\;\mathrm{freq}.}}>0.2,\\ R_{\mathrm{neg}} & = & \frac{\sum F{\left(sIQ\right)}_{f<0}}{\sum F{\left(sIQ\right)}_{\mathrm{all}\;\mathrm{freq}.}}>0.25 \end{array}$$

(14) $$|g_{1}\left(1\right)|>0.2$$

where F denotes the Fourier transform. These criteria skipped the poor quality data, which also greatly reduced the processing time.

Then, the fitting procedure was applied for both *sIQ*_neg_ and *sIQ*_pos_, respectively. In practice, random noise results in a prompt “drop” of *g*_1_(1), i.e., the change of *g*_1_(0) to *g*_1_(1) is not a smooth transition compared to *g*_1_(1) to the end of the decorrelation as the noise is uncorrelated. Therefore, the *g*_1_(*τ*) equation was modified by using an “F” factor to account for this “drop.” Also, it is worth noting that when using a linear transducer array the ultrasound PSF is anisotropic in the transverse directions, i.e., *σ*_*x*_ ≠ *σ*_*y*_. In the present experimental setup, *σ*_*y*_ was more than three times larger than *σ*_*x*_ which results in a more than nine times slower signal decorrelation rate from *v*_*y*gp_ compared to that from *v*_*x*gp_. Therefore, the *y* component was omitted from the *g*_1_(*τ*) fitting to simplify the data processing. In addition, in the case of Gaussian velocity distribution, *σ*_*vz*_ is proportional to the maximum speed in the center line and also linearly related to the group velocity *v*_*z*gp_. Thus, *σ*_*vz*_ in Equation (3) can be replaced with *σ*_*vz*_ =  *p* · *v*_*z*gp_ where *p* is a linear factor with a range of [0 1]. Thus, the theoretical *g*_1_(*τ*) model used for fitting the experimental data is

(15) $$g_{1}\left(\tau \right)=F\cdot e^{-\frac{{\left(v_{x\mathrm{gp}}\tau \right)}^{2}}{4\sigma_{x}^{2}}-\frac{{\left(v_{z\mathrm{gp}}\tau \right)}^{2}}{4\sigma_{z}^{2}}}e^{-{\left(p\cdot v_{z\mathrm{gp}}\cdot k_{0}\cdot \tau \right)}^{2}}e^{i2k_{0}\tau v_{z\mathrm{gp}}}$$

where *F* represents the correlated dynamic fraction which accounts for the *g*_1_(*τ*) value drop at the first time lag due to uncorrelated signal fluctuations (e.g., noise); *v_x_* and *v_z_* are the flow speed in the *x* and *z* directions respectively; *σ*_*vz*_ =  *p* · *v_z_* accounts for the speed distribution within the measurement voxel where *p* is a linear factor with a range of [0 1]; *σ*_*x*_ and *σ*_*z*_ are the US voxel Gaussian profile width at the 1/*e* value of the maximum intensity of the point spread function (PSF) in the *x* and *z* directions, respectively; and *k*_0_ = 2*π*/*λ*_0_  is the wave number of the central frequency of the transducer.

A proper initial guess of the unknown parameters (i.e., F, *v*_*x*gp_, *v*_*z*gp_, and *p*) is important to achieve high fitting accuracy and efficiency. The initial guess of *F*_0_ was set to be *F*_0_ = |*g*_1_(1)|. As the axial movement caused the phase change of *g*_1_(*τ*), the phase information of *g*_1_(*τ*) was used to determine *v*_*z*gp0_ by finding the time lag *τ*_*V*_ when *g*_1_(*τ*) reaches the first minimum

(16) $$v_{z\mathrm{gp}0}=\frac{\lambda_{0}}{4\tau_{V}}\;$$

A mesh of *v*_*x*gp_ and *p* values was tested to determine the initial guess of *v*_*x*gp0_ and *p*_0_ by finding the pair of *v*_*x*gp0_ and *p*_0_ that maximizes the coefficient of determination, *R*. *R* is defined in Equation (17) and was also used in the final fitting process as the objective function for a constrained least squares regression nonlinear fitting procedure to estimate the values for *F*, *v*_*x*gp_, *v*_*z*gp_, and *p* based on the initial guesses

(17) $$\begin{array}{c} R\;=\;1\;-\;\frac{〈{\left|g_{1_{\exp}}\left(\tau \right)\;-\;\left(F\;\cdot \;e^{-\frac{{\left(v_{x\mathrm{gp}}\tau \right)}^{2}}{4\sigma_{x}^{2}}\;-\;\frac{{\left(v_{z\mathrm{gp}}\tau \right)}^{2}}{4\sigma_{z}^{2}}}e^{-{\left(p\cdot v_{z\mathrm{gp}}\cdot k_{0}\cdot \tau \right)}^{2}}e^{i2k_{0}\tau v_{z\mathrm{gp}}}\right)\right|}^{2}〉}{{\left\langle |g_{1_{\exp}}\left(\tau \right)-\left\langle g_{1_{\exp}}\left(\tau \right)\right\rangle |\right\rangle }^{2}} \end{array}$$

where $g_{1_{\exp}}\left(\tau \right)$ is the experimental *g*_1_(*τ*) calculated with Equation (2); 〈…〉 indicates temporal ensemble averaging; and |…| indicates the absolute value.

Finally, the axial and total velocity maps were obtained for both descending and ascending flows, as shown in Figure S1e (Supporting Information).

### 4.3 Power Doppler‐fUS and Color Doppler‐fUS Calculation

The PD‐fUS image was calculated as^[^ ^4^ ^]^

(18) $$PDI=\frac{1}{N}\;\sum_{i=1}^{N}sIQ^{2}\left(t_{i}\right)$$

where *N* is the number of samples and *sIQ* is the complex ultrasound quadrature signal of the moving particles.

The axial velocity based on the conventional Color Doppler calculation is obtained with^[^ ^10^ ^]^

(19) $$v_{cz}=-\frac{c}{2f_{0}}\frac{\int_{-f_{s}/2}^{f_{s}/2}f\cdot {\left|F(sIQ)\right|}^{2})df}{\int_{-f_{s}/2}^{f_{s}/2}{\left|F(sIQ)\right|}^{2})df}$$

where *c* is the sound speed in the medium and *c* = 1540 m s^−1^ was used in this study; *f*_0_ is the transducer center frequency; *f_s_* is the frame rate; and F denotes the Fourier transform.

Further, for a fair comparison with vUS which obtains velocity map based on the directional filtered data (*sIQ*_neg_ and *sIQ*_pos_), Color Doppler was used to process the same directional filtered data to obtain descending and ascending speeds (Figure 6c1)

(20) $$\begin{array}{ccc} v_{cz,\mathrm{dsnd}} & = & -\frac{c}{2f_{0}}\frac{\int_{-f_{s}/2}^{0}f\cdot {\left|F(sIQ)\right|}^{2})df}{\int_{-f_{s}/2}^{0}{\left|F(sIQ)\right|}^{2})df},\\ v_{cz,\mathrm{asnd}} & = & -\frac{c}{2f_{0}}\frac{\int_{0}^{f_{s}/2}f\cdot {\left|F(sIQ)\right|}^{2})df}{\int_{0}^{f_{s}/2}{\left|F(sIQ)\right|}^{2})df} \end{array}$$

### 4.4 Ultrasound Localization Microscopy

The ultrasound localization microscopy (ULM) images and the ULM‐based velocity maps (vULM) were obtained based on a microbubble tracking and accumulation method described in refs. [13, 14]. Briefly, a frame‐to‐frame subtraction was applied to the IQ data to get the dynamic microbubble signal. The images of the microbubble were rescaled to have a pixel size of 10 µm ×  10 µm. The centroid position for each microbubble was then identified with 10 µm precision by deconvolving the system point spread function. By accumulating the centroid positions over time, a high‐resolution image of the cerebral vasculature image (ULM) was obtained. Further, by identifying and tracking the same microbubble's position, the in‐plane flow velocity of the microbubble could be calculated based on the travel distance and the imaging frame rate. The final velocity for coordinates (*z, x*) consisted of descending and ascending flows, and the speed for each direction was obtained by averaging the same directional flow speed at all time points when the absolute value was greater than 0, respectively.

### 4.5 Numerical Simulation

In this study, 2D (*x*–*z*) flow and ultrasound detection was simulated to validate vUS. Point scattering particles (5 µm in diameter) were randomly generated at the initialization segment which was outside the ultrasound measurement voxel. Then the flowing positions were calculated for all time points based on the preset flow speed and flow angle at a temporal rate of 5 KHz. The detected ultrasound signal (*sIQ*) was obtained based on Equation (1) for each time point. Then the simulated *g*_1_(*τ*) was calculated according to Equation (2) with 1000 observation time points (i.e., 200 ms) and 100 autocorrelation calculation time lags (i.e., 20 ms). Flow velocity was then reconstructed by applying vUS processing on the simulated *g*_1_(*τ*).

### 4.6 Phantom Experiment and Data Processing

For the phantom validation experiment, a plastic microtube (inner diameter 580 µm, Intramedic Inc.) was buried in a homemade agar phantom with an angle of ≈30° (angled flow), and another plastic microtube was aligned close to ≈0° (transvers flow) in another homemade agar phantom. A blood solution was pumped through the tubes with a syringe pump (Harvard Apparatus) at speeds of 1, 3, 5, 7, 9, 11, 13, 15, 20, 25, and 30 mm s^−1^. SVD was performed to filter the background signal clutter by removing the first two highest singular value components. Since the diameter of the tube was much larger than the ultrasound resolution, the red blood cell speed distribution could be considered uniform. Therefore, the linear value *p* in Equation (15) was set to 0 (i.e., *σ*_*vz*_ =  0) for the phantom data processing.

### 4.7 Animal Preparation

The animal experiments were conducted following the Guide for the Care and Use of Laboratory Animals, and the experiment protocol was approved by the Institutional Animal Care and Use Committees of Boston University.

In this study, 12–16‐week old C57BL/6 mice (22–28 g, male, Charles River Laboratories) were used. Animals were housed under diurnal lighting conditions with free access to food and water. Mice were anesthetized with isoflurane (3% induction, 1–1.5% maintenance, in 1 L min^−1^ oxygen) while the body temperature was maintained with a homeothermic blanket control unit (Kent Scientific) during surgery and anesthetized imaging sessions. After removal of the scalp, a custom‐made PEEK headbar was attached to the skull using dental acrylic and bone screws. The skull between lambda and bregma extending to temporal ridges was removed as a strip. A PMP film cut to the size of the craniotomy was then secured to the skull edges. Since the PMP was flexible, brain was protected by a cap attached to the head bar. The animal was allowed to recover for 3 weeks before the imaging sessions. During surgery and anesthetized imaging, heart rate and oxygen saturation were noninvasively monitored (Mouse Stat Jr, Kent Scientific) and all noted measurements were within the expected physiological range. For awake imaging, animals were trained to be head fixed for at least two weeks before the imaging session using sweetened condensed milk as treat. According to the optical coherence tomography measurement, the lateral and transverse motion due to respiratory and cardiac pulse was smaller than 1–2 µm, which was significantly smaller than the resolution ability of the ultrasound system (≈100 µm). Therefore, the experimental setup could largely minimize the confounding factor due to bulk motion.

### 4.8 In Vivo Experiment and Data Processing

#### 4.8.1 Experimental Setup

Agarose phantom (no scattering) was used to fill the cranial window, which serves as the acoustic matching medium between a water container and the mouse brain. The bottom of the water container was covered with a thin clear film preventing water leakage. To maintain the brain temperature of experimental animal, degassed warm water (37° ± 1°) was circulating through the water container and, along with the agarose phantom, worked as the acoustic transmitting medium between the ultrasound transducer and the mouse brain, as shown in Figure 4a. An anteroposterior linear translating stage was used to carry the ultrasound probe to acquire data at different coronal planes.

For anesthetized imaging, the experimental animal was anesthetized by isoflurane through a nose cone while the body temperature was maintained at 37° with a homeothermic blanket control unit (Harvard Apparatus) and its head was fixed by a stereotaxic frame. For awake imaging, the experimental animal head was fixed by attaching the headbar to a customized mount and the animal was treated with milk every ≈30 min.

#### 4.8.2 In Vivo Validation

For in vivo validation, animals were anesthetized with isoflurane and the body temperature was maintained at 37°. vUS data were first acquired at different coronal planes and followed by microbubble injection for ULM/vULM imaging for each coronal plane. 0.03 mL commercial microbubble suspension (5.0–8.0 × 10^8^ microbubbles mL^−1^, Optison, GE Healthcare, Milwaukee, WI, USA) was administered through retro‐orbital injection of the mouse eye. The vULM map was rescaled to have the same pixel size (25 µm × 25 µm) as vUS map. For a fair comparison, both the vULM and the vUS measurements were applied with a spatial mask that ensures nonzero valued pixels for both vUS and vULM measurements.

#### 4.8.3 Whisker Stimulation

*N* = 3 mice were trained and used for the whisker stimulation experiment. An air puffer machine (Picospritzer III, Parker Inc.) was used for the whisker stimulation experiments. The outlet of the air tube was placed ≈15 mm behind the whiskers. Two stimulation patterns were used in this study: the first stimulation pattern (Figure 4 and Figure S6a, Supporting Information) consisted of 30 s baseline and followed by 10 trials of 15 s stimulation and with a 45 s interstimulus interval, and the second stimulation pattern (Figure S6b, Supporting Information) consisted of 20 s baseline and followed by 10 trials of 5 s stimulation and with a 25 s interstimulus interval. A motion correction method was used to replace the signal value at strong motion time points with the median value of adjacent time points. The stimulation frequency was 3 Hz.

The whisker stimulation activation maps were calculated as the correlation coefficient *r* between the blood flow velocity *v*(*z*,*x*,*t*) and the temporal stimulus pattern *S*(*t*)

(21) $$r\left(z,x\right)=\frac{\sum_{t=1}^{N}\left(v\left(z,x,t\right)-\bar{v\left(z,x\right)}\right)\left(S\left(t\right)-\bar{S}\right)}{\sqrt{\sum_{t=1}^{N}{\left(v\left(z,x,t\right)-\bar{v\left(z,x\right)}\right)}^{2}}\sqrt{\sum_{t=1}^{N}{\left(S\left(t\right)-\bar{S}\right)}^{2}}}$$

where

(22) $$\bar{v(z,x)}=\frac{1}{N}\;\sum_{t=1}^{N}v\left(z,x,t\right)\;\mathrm{and}\;\bar{S}=\frac{1}{N}\;\sum_{t=1}^{N}S\left(t\right)$$

where *N* is the total acquisition. The correlation coefficient was transformed to *z* score according to Fisher's transform (Equation 16) and the level of significance was chosen to be *z >* 4.43 (*p* < 0.001, one‐tailed test), which corresponds to *r* > 0.2

(23) $$z=\frac{\sqrt{N-3}}{2}\cdot \ln\frac{1+r}{1-r}$$

## Conflict of Interest

The authors declare no conflict of interest.

## Authors Contributions

J.T. and D.A.B. conceived of the technology and designed this study. J.T., D.D.P., T.L.S., and D.A.B. developed the theoretical model and analyzed the results. J.T. derived the theoretical formula, developed the data processing method, constructed the experimental setup, carried out experiments, and wrote the manuscript. K.K., E.E., and B.L. developed the surgical protocol for chronic imaging on awake mice, carried out animal experiments, and analyzed the results. J.T.G designed the head bar. D.A.B. supervised this study. All authors discussed the results and contributed to the final version of the manuscript.

## Supporting information

Supporting Information

Supplemental Video 1
