# Supplementary material for: Functional Ultrasound Speckle Decorrelation‐Based Velocimetry of the Brain
Source: Adv Sci (Weinh). 2020 Jul 26;7(18):2001044. doi: 10.1002/advs.202001044 (PMC7509671; doi:10.1002/advs.202001044)
Supplement: Supplementary file 1 — Supporting Information [file ADVS-7-2001044-s001.pdf]

Copyright WILEY-VCH Verlag GmbH & Co. KGaA, 69469 Weinheim, Germany, 2020.

ADVANCED SCIENCE
Open Access

Supporting Information

for *Adv. Sci.,* DOI: 10.1002/advs.202001044

Functional Ultrasound Speckle Decorrelation-Based Velocimetry of the Brain

*Jianbo Tang, Dmitry D. Postnov, Kivilcim Kilic, Sefik Evren Erdener, Blaire Lee, John T. Giblin, Thomas L. Szabo, and David A. Boas**

Copyright WILEY-VCH Verlag GmbH & Co. KGaA, 69469 Weinheim, Germany, 2020.

Supporting Information

Functional ultrasound speckle decorrelation-based velocimetry of the brain

Jianbo Tang[1], *Dmitry D. Postnov*[1,3], *Kivilcim Kilic*[1], *Sefik Evren Erdener*[1], *Blaire Lee*[1], *John T. Giblin*[1], *Thomas L. Szabo*[1] *& David A. Boas*[1,*].

I. Supplementary Figures

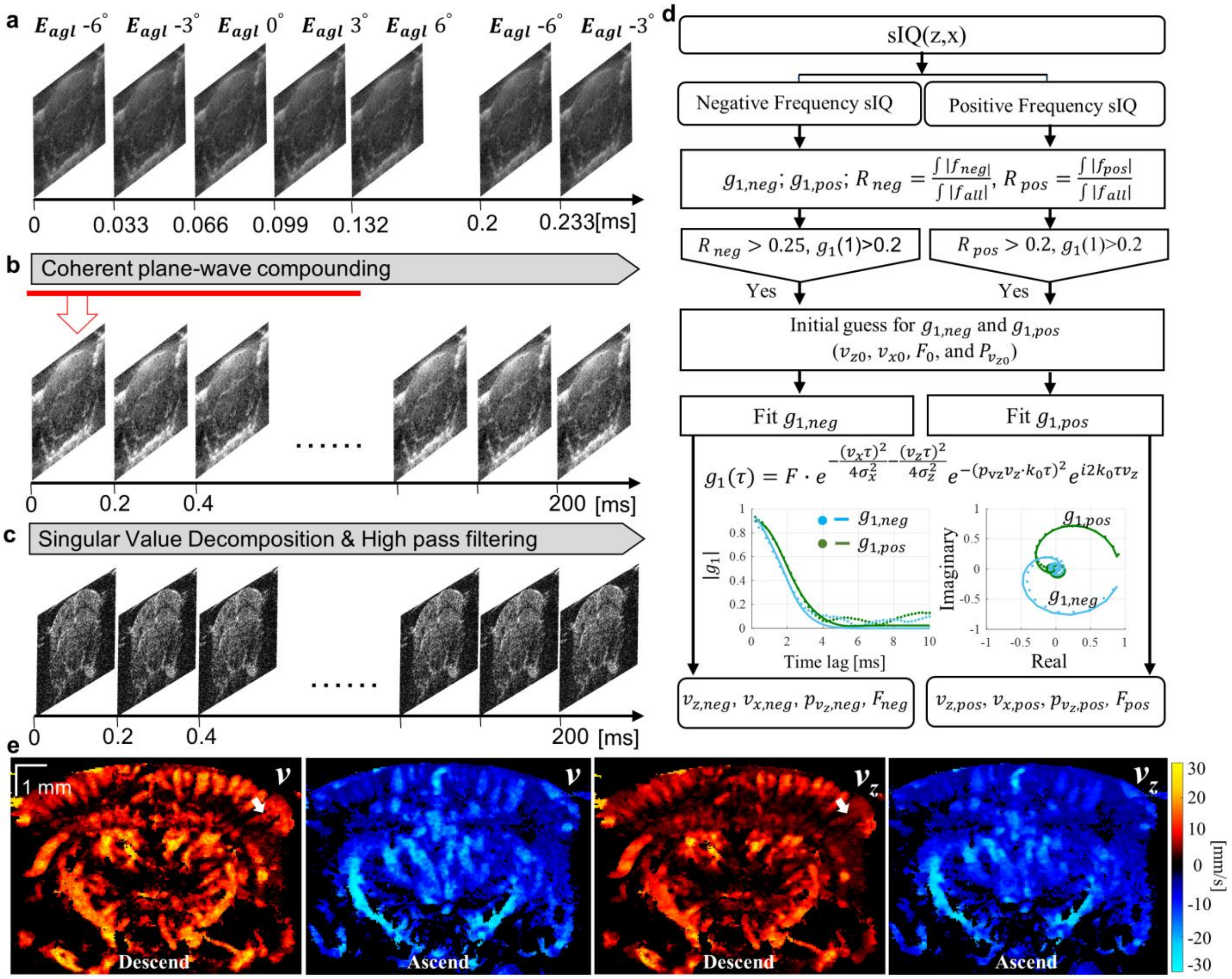

Figure S1 | vUS implementation and data processing. (a) Ultrasound pulse & acquisition sequence. (b) Coherent plane-wave compounding were performed on the 5 tilted emission angle frames and produced a compounded image at a frame rate of 5 kHz. (c) Clutter rejection were performed to remove static background and bulk motion signal components. (d) Negative and positive frequency components of a measurement voxel are processed separately for *in vivo* data vUS processing; dots: experimental data; solid lines: fitting results. (e) Descending and ascending blood flow velocity maps reconstructed by vUS of a coronal plane (~Bregma -2.18 mm) of a mouse brain.

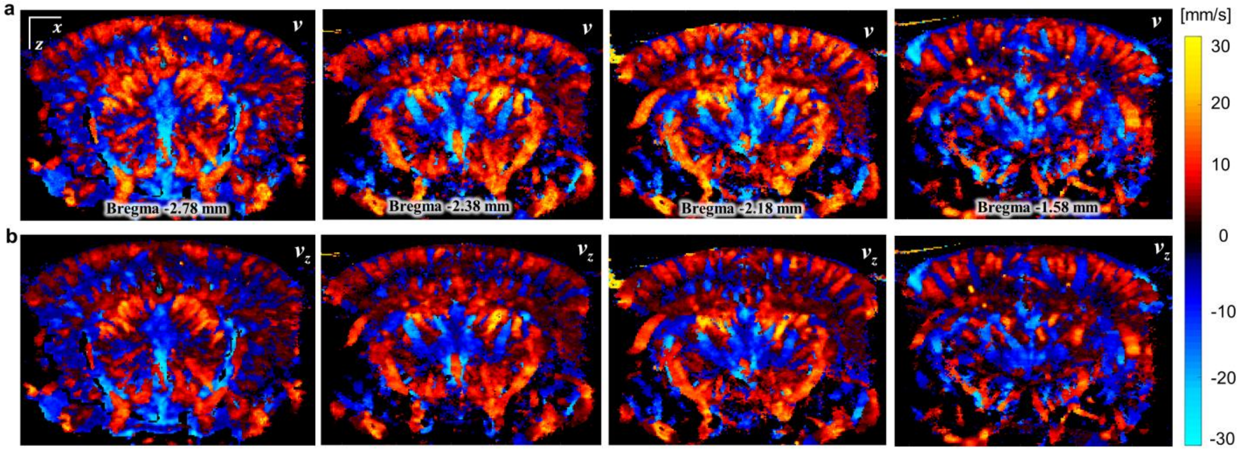

Figure S2 | Total velocity (a) and axial velocity (b) obtained with vUS at different coronal planes of a mouse brain. Descending flow velocity map was overlapped on ascending flow velocity map.

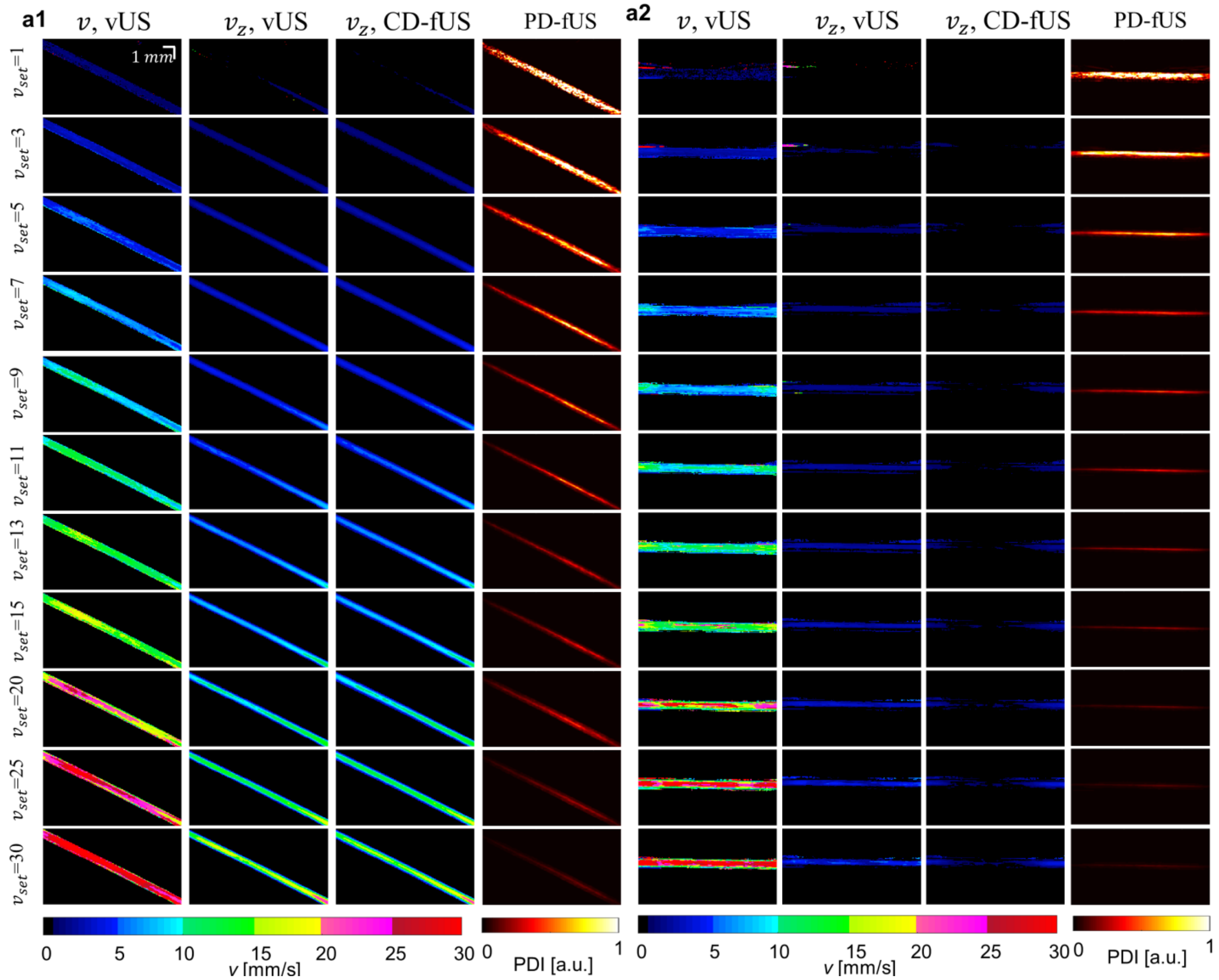

Figure S3 | Phantom experiment validation and comparison. (a) Results for angled flow phantom experiments. (b) Results for transverse flow phantom experiments. vUS is able to accurately measure both axial and transverse velocity components while CD-fUS is not capable of measuring the transverse flow velocity component. In addition, vUS is able to accurately differentiate the axial velocity component from the transverse velocity component given its ability to determine flow direction. Compared to PD-fUS, vUS measured velocity has a linear relationship with the preset speeds, while the PD-fUS measured signal decreases nonlinearly with increasing preset speed.

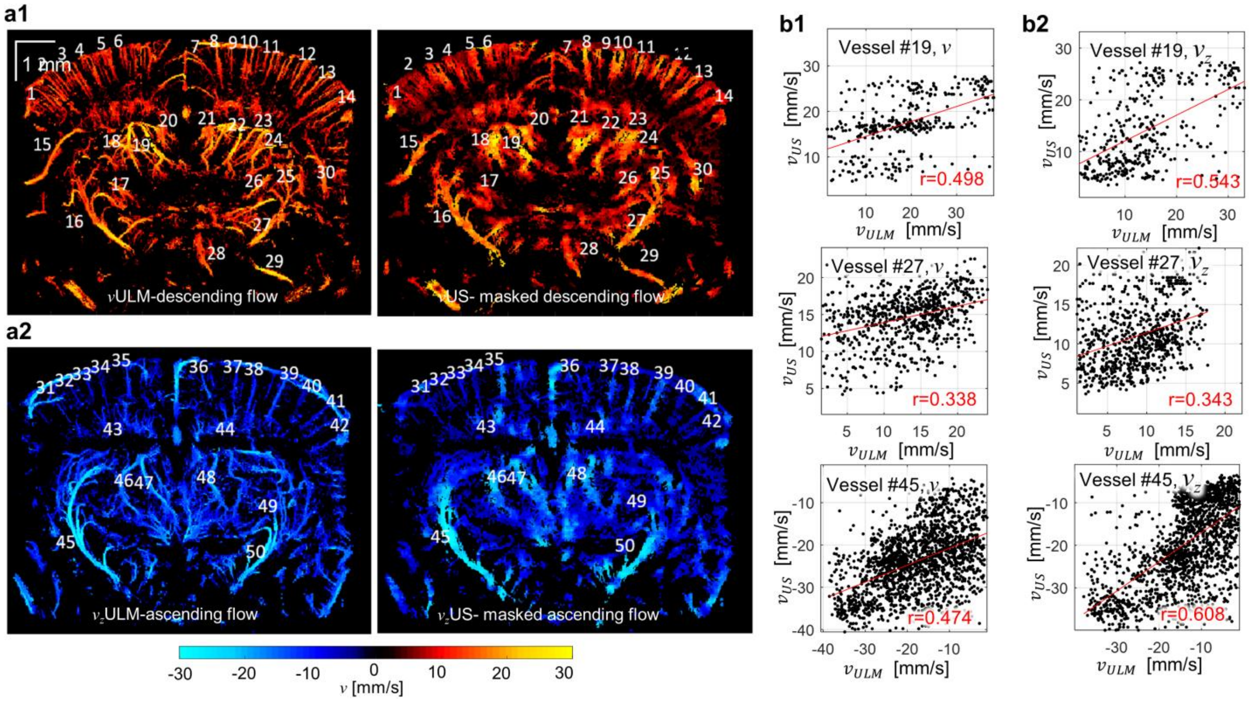

Figure S4 | *in vivo* validation by comparing vUS with vULM. (a) The numbers show the indices of selected vessel for vessel-to-vessel comparison between vUS and vULM. (b1) Scatter plots of total velocity of three representative vessels show the pixel-to-pixel correlation between vULM and vUS. (b2) Scatter plots of axial velocity of three representative vessels show the pixel-to-pixel correlation between vULM and vUS.

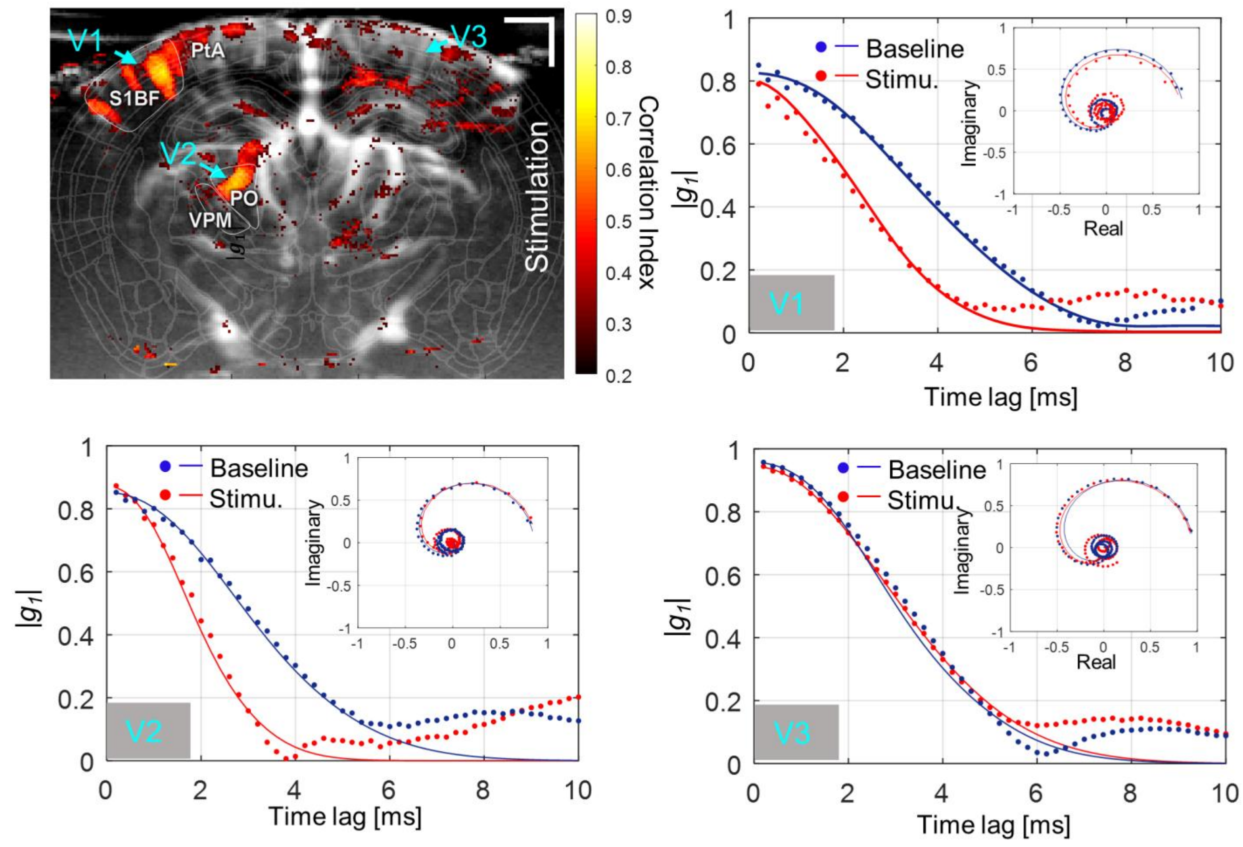

Figure S5 | $g_1(\tau)$ decorrelation of the same spatial location (measurement voxel) in vessels of V1, V2, and V3 during baseline and whisker stimulation states. $g_1(\tau)$ decays faster during whisker stimulation compared to baseline in the responding vessels of V1 and V2, while they have similar decorrelation rate in the control vessel of V3.

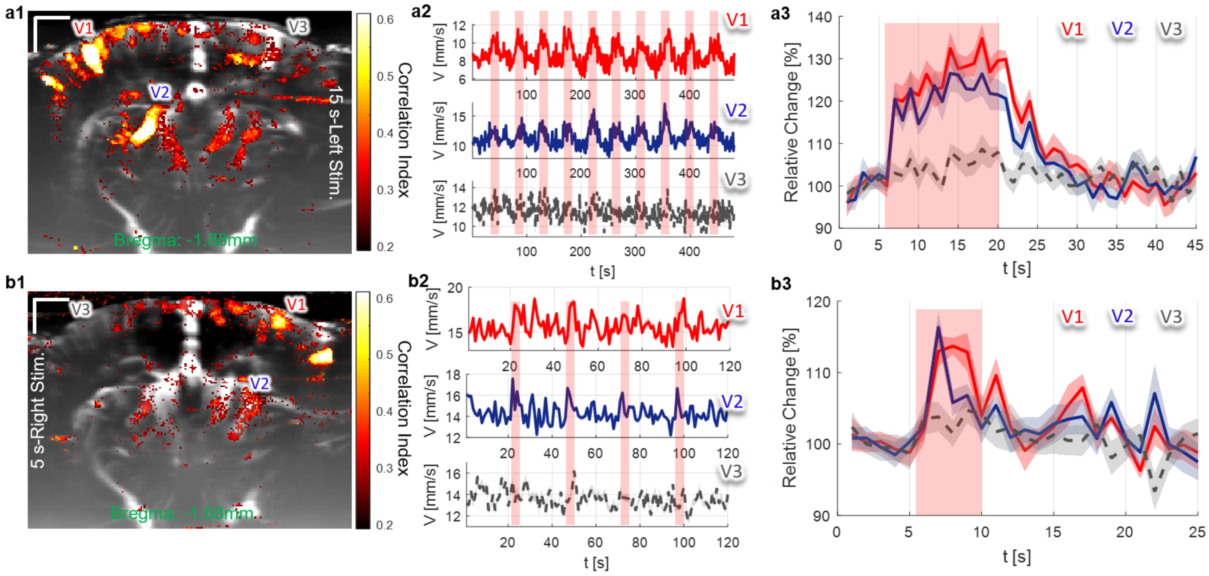

Figure S6 | Representative whisker stimulation results. (a) Results of 15 seconds left side whisker stimulation; (a1) Activation map; (a2) Blood flow velocity time courses for the three vessels marked in (a1); (a3) 10 trials averaged relative response of the three vessels. (b) Results of 5 seconds right side whisker stimulation at Bregma ~ -1.58 mm; (b1) Activation map; (b2) Blood flow velocity time courses for the three vessels marked in (b1); (b3) 10 trials averaged relative response of the three vessels.

II. Function description for vUS data processing

Note: vUS data processing code and example data is available from: **Supplementary Code**.

A. vUS data processing for *in vivo* data

A.1. main function

```
%% IQ to vUS data processing for in vivo experiment
clear all; clc
addpath('./SubFunctions/');
%% Use GPU calculation or not
useGPU = questdlg('Use GPU for data processing?', 'Select', ...
    'YES', 'NO', 'Cancel', 'Cancel');
%% Load data
disp(['Loading data, ', datestr(datetime('now'))]);
load ('./DATA/invivoData.mat');
[nz,nx,nt]=size(IQ);
PRSSinfo.g1StartT=1;
PRSSinfo.g1nT=nt;
PRSSinfo.g1nTau=100;
PRSSinfo.rFrame=5000; % sIQ frame rate, Hz
PRSSinfo.SVDrank=[25, nt];
PRSSinfo.HPfC=25; % high pass frequency cutoff
PRSSinfo.FWHM=[125 100]*1e-6;  % (X, Z) spatial resolution, Full Width at Half Maximum of point spread function, m
PRSSinfo.C=1540;                   % sound speed, m/s
PRSSinfo.f0=16.625*1e6;              % Transducer center frequency, Hz
PRSSinfo.xCoor=interp(P.xCoor,PRSSinfo.rfnScale);
PRSSinfo.zCoor=interp(P.zCoor,PRSSinfo.rfnScale);
PRSSinfo.MpVz=1; % maximum p value for SigmaVz
PRSSinfo.NEQ=0; % no noise equalization
%% 1. Clutter rejection
disp(['Clutter Rejection - ', datestr(datetime('now'))]);
[sIQ, sIQHP, sIQHHP, eqNoise]=IQ2sIQ(IQ(:,:,1:PRSSinfo.g1nT),PRSSinfo); % 0: no noise equalization
[nz,nx,nt]=size(sIQ);
clear IQ
%% 2. vUS data processing
disp(['g1-based vUS Processing - ', datestr(datetime('now'))]);
if strcmp(useGPU, 'YES')
    disp('GPU-based vUS Processing ...(NOTE: it taks around 30 seconds)');
    tic;[F, Vz, V,  pVz, R]=sIQ2vUS_NPDV_GPU(sIQ, PRSSinfo);toc
else
    disp('CPU-based vUS Processing ...(NOTE: it taks around 400 seconds)');
    tic;[F, Vz, V,  pVz, R]=sIQ2vUS_NPDV(sIQ, PRSSinfo);toc
end
%% 3. save results and plot V and Vz
[VzCmap]=Colormaps_fUS;
save(['./vUS.mat'],'-v7.3','F','Vz','V','R','pVz');
disp(['Results are saved! - ', datestr(datetime('now'))]);
% figure plot
Coor.x=PRSSinfo.xCoor;     Coor.z=PRSSinfo.zCoor;
Fig=figure;
set(Fig, 'Position',[300 400 1300 400]);
subplot(1,2,1)
Fuse2Images(V(:,:,1),V(:,:,2),[-30 30],[-30 30],Coor.x,Coor.z,2.5);
title(['vUS, V [mm/s]']);
subplot(1,2,2)
Fuse2Images(Vz(:,:,1),Vz(:,:,2),[-30 30],[-30 30],Coor.x,Coor.z,2.5);
title(['vUS, Vz [mm/s]']);
```

A.2. function IQ2sIQ

```
%% IQ to sIQ with SVD data processign, sIQ to sIQHP with high pass filtering on sIQ.
% Input:
    % IQ: complex IQ data, obtained with RF2IQ, [nz,nx,nt]
    % PRSSinfo.SVDrank: SVD rank [low high]
    % PRSSinfo.HPfC:  High pass filtering cutoff frequency, Hz
```

```
    % PRSSinfo.NEQ: do noise equalization? 0: no noise equalization; 1: apply noise equalization
    % PRSSinfo.rFrame: imaging frame rate, Hz
% output:
    % sIQ: SVD clutter rejected data, [nz,nx,nt]
    % sIQHP: SVD+HP clutter rejected data, [nz,nx,nt], cutoff frequency: PRSSinfo.HPfC
    % sIQHHP: SVD+HHP clutter rejected data, [nz,nx,nt], cutoff frequency: 70 Hz
% subfunction:
    % [sIQ, Noise]=SVDfilter(IQ,SignalRank)
function [sIQ, sIQHP, sIQHHP, eqNoise]=IQ2sIQ(IQ,PRSSinfo)
```

A.3. function sIQ2vUS_NP_DV

```
%% US g1 fit for in vivo data, fit negative and postive frequency signal separately
% input:
    % sIQ: bulk motion removed data, [nz,nx,nt]
    % PRSSinfo: data processing parameters, including
        % PRSSinfo.FWHM: (X, Y, Z) spatial resolution, Full Width at Half Maximum of point spread function, m
        % PRSSinfo.rFrame: sIQ frame rate, Hz
        % PRSSinfo.f0: Transducer center frequency, Hz
        % PRSSinfo.C: Sound speed in the sample, m/s
        % PRSSinfo.g1nT: g1 calculation sample number
        % PRSSinfo.g1nTau: maximum number of time lag
        % PRSSinfo.SVDrank: SVD rank [low high]
        % PRSSinfo.HPfC:  High pass filtering cutoff frequency, Hz
        % PRSSinfo.NEQ: do noise equalization? 0: no noise equalization; 1: apply noise equalization
        % PRSSinfo.rfnScale: spatial refind scale
        % PRSSinfo.MpVz: maximu pVz
        % PRSSinfo.useMsk: 1: use ULM data as spatial mask; 0: no spatial mask
        % PRSSinfo.ulmMsk: ULM-based spatial constrain mask
% output:
    % F: dynamic component fraction, [nz,nx,2], 2: [real,imag]
    % Vz: axial-direction velocity component, [nz,nx], mm/s
    % V=sqrt(Vx.^2+Vz.^2), [nz,nx], mm/s
    % pVz: Vz distribution (sigma-Vz), [nz,nx]
    % R: fitting accuracy, [nz,nx]
function [F, Vz, V, pVz, R]=sIQ2vUS_NPDV_GPU(sIQ, PRSSinfo)
function [F, Vz, V, pVz, R]=sIQ2vUS_NPDV(sIQ, PRSSinfo)
```

B. vUS data processing (SV model) for phantom data

B.1. Main function

```
%% IQ to vUS data processing for ex vivo data using the basic model
clear all; clc
addpath('./SubFunctions');
%% Use GPU calculation or not
useGPU = questdlg('Use GPU for data processing?', 'Select', ...
    'YES', 'NO', 'Cancel', 'Cancel');
%% Load data
disp(['Loading data, ', datestr(datetime('now'))]);
% load ('./DATA/phantomData5a.mat');  % angled flow, preset speed 5 mm/s
load ('./DATA/phantomData15a.mat');  % angled flow, preset speed 15 mm/s
% load ('./DATA/phantomData9t.mat');  % transverse flow, preset speed 9 mm/s
% load ('./DATA/phantomData25t.mat');  % transverse flow, preset speed 25 mm/s
% IQ: beamformed complex quadratue data
[nz,nx,nt]=size(IQ);
PRSSinfo.g1StartT=1;
PRSSinfo.g1nT=nt;
PRSSinfo.g1nTau=100;
PRSSinfo.rFrame=5000; % sIQ frame rate, Hz
PRSSinfo.SVDrank=[3, nt];
PRSSinfo.HPfC=25; % high pass frequency cutoff
PRSSinfo.FWHM=[125 100]*1e-6;  % (X, Z) spatial resolution, Full Width at Half Maximum of point spread function, m
PRSSinfo.C=1540;                    % sound speed, m/s
PRSSinfo.f0=16.625*1e6;             % Transducer center frequency, Hz
PRSSinfo.rfnScale=1;
PRSSinfo.xCoor=interp(P.xCoor,PRSSinfo.rfnScale);
PRSSinfo.zCoor=interp(P.zCoor,PRSSinfo.rfnScale);
PRSSinfo.NEQ=0; % no noise equalization
%% Clutter rejection
disp(['Clutter Rejection - ', datestr(datetime('now'))]);
[sIQ, sIQHP, sIQHHP, eqNoise]=IQ2sIQ(IQ(:,:,1:PRSSinfo.g1nT),PRSSinfo); % 0: no noise equalization
[nz,nx,nt]=size(sIQ);
clear IQ
disp(['Power Doppler Processing - ', datestr(datetime('now'))]);
[PDI]=sIQ2PDI(sIQ);  % PDI processing
disp(['Color Doppler Processing - ', datestr(datetime('now'))]);
Vcz0=(ColorDoppler(sIQ,PRSSinfo)); % color Doppler, all frequency
disp(['g1-based vUS Processing - ', datestr(datetime('now'))]);
if strcmp(useGPU, 'YES')
    Dev=gpuDevice;
    disp('GPU-based vUS Processing ...(NOTE: it taks around 4 seconds)');
    tic;[F, Vz, Vx, V, R]=sIQ2vUS_SV_GPU(sIQ, PRSSinfo);toc
else
    disp('CPU-based vUS Processing ...(NOTE: it taks around 30 seconds)');
    tic;[F, Vz, Vx, V, R]=sIQ2vUS_SV(sIQ, PRSSinfo);toc
end
Vcz=imresize(Vcz0, [nz,nx]*PRSSinfo.rfnScale,'bilinear').*CR;
save(['./vUS.mat'],'-v7.3','F','Vz','Vx','V','Vcz','R','PRSSinfo','P');
disp(['Results are saved! - ', datestr(datetime('now'))]);
%% figure plot
[VzCmap,VzCmapDn, VzCmapUp, PhtmCmap]=Colormaps_fUS;
Coor.x=[1:nx]*0.05/PRSSinfo.rfnScale;
Coor.z=[1:nz]*0.05/PRSSinfo.rfnScale;
Fig=figure;
set(Fig,'Position',[400 400 1700 350])
subplot(1,3,1)
h1=imagesc(Coor.x,Coor.z,abs(V));
colormap(PhtmCmap);
caxis([0 30]);
colorbar
axis equal tight;
xlabel('x [mm]')
ylabel('z [mm]')
title('vUS-V [mm/s]')

subplot(1,3,2)
h2=imagesc(Coor.x,Coor.z,abs(Vz));
colormap(PhtmCmap);
```

```
caxis([0 30]);
colorbar
axis equal tight;
xlabel('x [mm]')
ylabel('z [mm]')
title('vUS-Vz [mm/s]')

subplot(1,3,3)
h3=imagesc(Coor.x,Coor.z,abs(Vcz));
colormap(PhtmCmap);
caxis([0 30]);
colorbar
axis equal tight;
xlabel('x [mm]')
ylabel('z [mm]')
title('Color Doppler-Vz [mm/s]')
```

B.2. function sIQ2vUS_SV

```
%% US g1 fit, fit all frequency signal, for single flow direction scenario

% input:
   % sIQ: bulk motion removed data
   % PRSSinfo: data acquistion information, including
      % PRSSinfo.FWHM: (X, Y, Z) spatial resolution, Full Width at Half Maximum of point spread function, m
      % PRSSinfo.rFrame: sIQ frame rate, Hz
      % PRSSinfo.f0: Transducer center frequency, Hz
      % PRSSinfo.C: Sound speed in the sample, m/s
      % PRSSinfo.g1nT: g1 calculation sample number
      % PRSSinfo.g1nTau: maximum number of time lag
      % PRSSinfo.SVDrank: SVD rank [low high]
      % PRSSinfo.HPfC:  High pass filtering cutoff frequency, Hz
      % PRSSinfo.NEQ: do noise equalization? 0: no noise equalization; 1: apply noise equalization
      % PRSSinfo.rfnScale: spatial refind scale
      % PRSSinfo.MpVz=0; %
output:
    % F: dynamic factor
   % Vz: axial velocity component, mm/s
   % Vx, transverse velocity component, mm/s
   % V: total velocity, mm/s
    % R: fitting accuracy
    % CR: vUS data processing criteria mask

function [F, Vz, Vx, V, R, CR]=sIQ2vUS_SV_GPU(sIQ, PRSSinfo);toc
function [F, Vz, Vx, V, R, CR]=sIQ2vUS_SV(sIQ, PRSSinfo);toc
```

B.3. function ColorDoppler

```
%% color Doppler data processing to get axial blood flow velocity
% input:
   % sIQ: bulk motion removed data
   % PRSSinfo: data acquistion information, including
      % PRSSinfo.rFrame: sIQ frame rate, Hz
      % PRSSinfo.f0: Transducer center frequency, Hz
      % PRSSinfo.C: Sound speed in the sample, m/s
% output:
   % Vcz: axial velocity calculated with Color Dopler, mm/s
function [Vcz]=ColorDoppler(sIQ,PRSSinfo)
```
